# Origin Specific Genomic Selection: A Simple Process To Optimize the Favorable Contribution of Parents to Progeny

**DOI:** 10.1534/g3.120.401132

**Published:** 2020-05-19

**Authors:** Chin Jian Yang, Rajiv Sharma, Gregor Gorjanc, Sarah Hearne, Wayne Powell, Ian Mackay

**Affiliations:** * Scotland's Rural College (SRUC), Kings Buildings, West Mains Road, Edinburgh, EH9 3JG, UK,; ^†^ The Roslin Institute and Royal (Dick) School of Veterinary Studies, University of Edinburgh, Easter Bush, Edinburgh, EH25 9RG, UK,; ^‡^ CIMMYT, KM 45 Carretera Mexico-Veracruz, El Batan, 56237, Texcoco, Edo. De Mexico, Mexico, and; ^§^ IMplant Consultancy Ltd., Chelmsford, UK

**Keywords:** introgression, barley, maize, NAM, genomic selection, plant breeding, Genomic Prediction, GenPred, Shared Data Resources

## Abstract

Modern crop breeding is in constant demand for new genetic diversity as part of the arms race with genetic gain. The elite gene pool has limited genetic variation and breeders are trying to introduce novelty from unadapted germplasm, landraces and wild relatives. For polygenic traits, currently available approaches to introgression are not ideal, as there is a demonstrable bias against exotic alleles during selection. Here, we propose a partitioned form of genomic selection, called Origin Specific Genomic Selection (OSGS), where we identify and target selection on favorable exotic alleles. Briefly, within a population derived from a bi-parental cross, we isolate alleles originating from the elite and exotic parents, which then allows us to separate out the predicted marker effects based on the allele origins. We validated the usefulness of OSGS using two nested association mapping (NAM) datasets: barley NAM (elite-exotic) and maize NAM (elite-elite), as well as by computer simulation. Our results suggest that OSGS works well in its goal to increase the contribution of favorable exotic alleles in bi-parental crosses, and it is possible to extend the approach to broader multi-parental populations.

There is a general concern that the genetic base of elite varieties of many crops has become very narrow, diminishing the ability of the farming landscape to respond positively and quickly to new challenges. To continue to introduce novel, high value genetic variation into the elite gene pool, breeding programs can select from crosses between their germplasm and materials from plant genetic resources; including wild species, landraces, and improved germplasm that are unadapted to the target environment. In these exotic crosses, marker assisted selection and backcrossing can effectively track a limited number of QTL accounting for a large proportion of the genetic variation for traits such as disease resistances. For highly polygenic traits, the introgression of novel variation from exotic sources is more complex for a number of reasons. First, QTL mapping for polygenic traits is ineffective or may capture only a small proportion of the genetic variation. Second, the breeding scheme and population size needed to effectively pyramid many QTL are unmanageable. Third, loci at which the exotic lines carry a favorable allele are often linked in repulsion with loci at which the elite lines carry a favorable allele. Consequently, selection on segregating populations from elite-exotic crosses tends to select for the elite background and favorable contribution from the exotic may be lost through linkage drag, or equivalently through hitchhiking. Further, it can be difficult to phenotype adaptive traits, including yield, in populations with a high proportion of unadapted/exotic germplasm. For this reason, selection is often restricted to populations derived from the first or second backcross to the elite parent, in which the average exotic contribution is one quarter or one eighth. However, this practice increases further the risk of loss of favorable alleles introduced from the exotic parent.

To overcome these problems, additional generations of crossing among progeny prior to selection can be made to reduce repulsion linkage. Recurrent selection programs have also been proposed to increase the frequency of favorable alleles from both elite and exotic donors over several generations (Hallauer and Carena 2012). Bernardo (2009) proposed genomic recurrent selection starting in the F_2_ before deriving recombinant lines and found this to be more effective than the conventional practice of selecting among lines derived from the backcross to the elite parent. More recently, Gorjanc *et al.* (2016) proposed genomic selection on a population established among exotic accessions to increase the frequency of favorable alleles prior to making crosses between the elite and (improved) exotic population. In simulation, this reduced the loss of favorable alleles from exotic sources compared to direct crossing. However, there is a risk that selective effort is wasted in increasing favorable allele frequencies in the improved exotic pool that are already at high frequency among elite lines. Ru and Bernardo (2019 and 2020) proposed backcrossing favorable linkage groups instead of QTL from exotic parent into elite parent using soybean nested association mapping (NAM) data as an example. Recently, Allier *et al.* (2019) proposed treating parental genome contribution as a trait in its own right, and suggested index or truncation selection on this and agronomic traits as a means of reducing the loss of favorable exotic alleles. In addition, Allier *et al.* (2020) proposed a method to identify exotic candidates that can provide the most benefit in elite-exotic crosses through maximizing favorable contributions from exotic parents.

The problems associated with introgression programs for quantitative traits also manifest in mainstream breeding programs. In a cross between two elite inbred lines, the favorable alleles at loci determining a polygenic trait are unlikely to be distributed equally between the two parents. For genetic progress, descendant lines must be selected in which both parents contribute favorable alleles, since only then can the performance of descendants exceed that of the best parent. Assuming for simplicity that all gene effects are equal, the selected line must be fixed for more favorable alleles than the best parent. However, selection among progeny may still result in a disproportionate contribution from the genome of the best parent. For example, Fradgley *et al.* (2019) found it common for an elite wheat line to share over 80% of its genetic material with one of its two elite parents.

In this paper we propose a simple process to quantify and therefore control the favorable contribution of parents to progeny with a technique called Origin Specific Genomic Selection (OSGS). We achieve this by partitioning a genomic prediction equation into two components: the first component is the contribution from markers where the favorable allele is carried by the primary (often elite) parent and the second component is the contribution from markers where the favorable allele is carried by the secondary (often exotic) parent. We test this method by within-cross prediction in two NAM datasets. The first is the HEB-25 barley NAM of backcross derived lines from an elite variety (Barke) and 25 wild barleys (Maurer *et al.* 2015). The second is the maize NAM of F_2_ derived lines from crosses between the inbred B73 and 25 lines selected to sample diversity among elite maize germplasm (Yu *et al.* 2008). We validate our results by computer simulations and discuss the implications of our results for introgression and pre-breeding together with broader applications in plant breeding, including the use of OSGS in multi-parental populations.

## Materials and Methods

### Genomic Selection (GS) and Origin Specific Genomic Selection (OSGS)

The mixed linear model commonly used in the training population of genomic selection (GS) can be generalized as:

y=Xb+Wu+e, [1]

where y is a vector of observed trait values for each individual,

X is a design matrix associating fixed effects with trait observations,

b is a vector of fixed effects,

W is a design matrix associating marker effects with trait observations,

u is a vector of marker effects with an assumed distribution of $N\left(0,I\sigma_{u}^{2}\right)$,

e is a vector of residuals with an assumed distribution of$N\left(0,I\sigma_{e}^{2}\right)$.

Then, once the marker effects are estimated ($\hat{u}$), we can predict breeding values ($\hat{a}$) for genotyped individuals (even non-phenotyped) as:

$$\hat{a}=W\hat{u}.$$ [2]

In a bi-parental cross, provided marker data are available on the parents, marker regression coefficients $\hat{u}$ can be partitioned into those that pertain to the favored alleles of the primary parent ${\hat{u}}_{1}$ and those that pertain to favored alleles of the secondary parent ${\hat{u}}_{2}$ such that $\hat{u}={\hat{u}}_{1}+{\hat{u}}_{2}$. We define the primary parent as the better performing line, the elite parent in an introgression program. The prediction equation [2] can then be partitioned into:

$${\hat{a}}_{1}=W{\hat{u}}_{1},$$ [3]

$${\hat{a}}_{2}=W{\hat{u}}_{2},$$ [4]

and

$$\hat{a}={\hat{a}}_{1}+{\hat{a}}_{2},$$ [5]

where ${\hat{a}}_{1}$ is the contribution from the primary parent and analogously ${\hat{a}}_{2}$ is the contribution from the secondary parent.

Among any set of individuals, we can then select based on $\hat{a}$, or on any index of ${\hat{a}}_{1}$ and ${\hat{a}}_{2}$. Thus, we refer to the former method as GS and the latter method as OSGS. Table 1 provides a simple example of the computation of $\hat{a},{\hat{a}}_{1}$ and ${\hat{a}}_{2}$ in which three favorable alleles out of ten are contributed by the exotic parent.

**Table 1:** An example of OSGS for ten unlinked markers segregating among inbred lines derived from the F_2_ cross of an elite and exotic parent. At each marker, elite and exotic homozygotes are respectively coded -1 and +1. Negative regression coefficient indicates the increasing allele for the trait is carried by the elite parent and a positive coefficient that the increasing allele is carried by the exotic parent. Here, seven favorable alleles originate from the elite and three from the exotic parent. For each individual (ID1-5), the sum of the products of marker genotypes and regression coefficients gives an estimate of the total breeding value, $\overset{\wedge }{a}$. Totalling products over the first three and last seven markers partitions the breeding value into contribution respectively from the elite (${\overset{\wedge }{a}}_{1}$) and exotic (${\overset{\wedge }{a}}_{2}$) parent. For the coefficients given, the expected correlation between $\overset{\wedge }{a}$ and ${\overset{\wedge }{a}}_{1}$is 0.89 and between $\overset{\wedge }{a}$ and ${\overset{\wedge }{a}}_{2}$ is 0.45. The expected correlation between ${\overset{\wedge }{a}}_{1}\;\text{and}\;{\overset{\wedge }{a}}_{2}$ is zero, since the markers are not linked in this example

Marker	Favored allele	Regression coefficient	Marker genotypes				
			ID1	ID2	ID3	ID4	ID5
M1	Elite	−0.95	−1	1	1	−1	1
M2	Elite	−0.34	−1	−1	1	−1	−1
M3	Elite	−0.47	−1	1	−1	−1	1
M4	Elite	−0.11	−1	−1	−1	−1	1
M5	Elite	−0.49	1	1	−1	−1	1
M6	Elite	−0.63	1	−1	−1	1	1
M7	Elite	−1.24	−1	−1	1	1	−1
M8	Exotic	0.22	1	1	1	1	−1
M9	Exotic	0.38	−1	−1	1	1	−1
M10	Exotic	0.82	1	1	1	−1	1
Total breeding value ($\hat{a}$)			2.65	1.07	0.59	0.27	−0.85
Elite contribution (${\hat{a}}_{1}$)			1.99	0.41	−0.83	0.49	−1.07
Exotic contribution (${\hat{a}}_{2}$)			0.66	0.66	1.42	−0.22	0.22

### Data analysis

**Figure 1:**
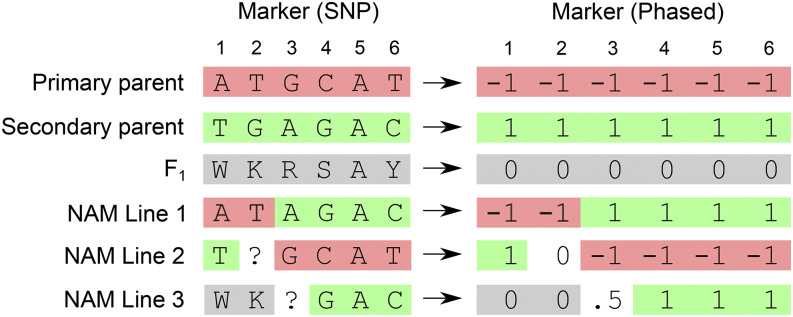
Example of recoding marker data for OSGS. SNP markers are originally coded according to standard IUPAC genetic codes (W = A/T, K = G/T, R = A/G, S = C/G, Y = C/T). In the phased markers, homozygous alleles from the primary parent are coded as -1, homozygous alleles from the secondary parent are coded as 1, and heterozygotes are coded as 0. Missing data and monomorphic SNPs are coded as the average between the two flanking, non-missing marker data.

No modification of an existing method for genomic prediction is required for OSGS provided the method estimates u. OSGS requires only that (i) allele origins are identified and (ii) marker estimates are partitioned into two classes with favorable alleles carried by the primary or by secondary parent. If marker genotypes are coded -1, 0, 1 with 0 as the heterozygous class (Figure 1), this partition is simply on the basis of the sign of the regression coefficients. Also, identifying allele origin is trivial in plant breeding scenarios with inbred parents.

Performance differences among various genomic prediction methods are generally minimal, especially if predictions are among closely related material over a limited number of generations (Daetwyler *et al.* 2013). In this paper therefore, we demonstrate only three standard methods: ridge regression (Hoerl and Kennard 1976) as implemented in rrBLUP (Endelman 2011), LASSO (Tibshirani 1996) as implemented in glmnet (Friedman *et al.* 2010), and BayesCπ (Habier *et al.* 2011) as implemented in BGLR (Pérez and de los Campos 2014) to test that OSGS is robust to the choice of method. All three methods are available as R packages and all analyses were performed with R 3.6.3 (R Core Team 2020).

In genomic selection, regression coefficients are typically estimated from one population of lines, the training or reference population, and the prediction equation is applied to a set of candidates with no trait information. Here, the emphasis is different since we are primarily interested in partitioning the observed phenotype of individuals into contributions from the two parents. As such, the question is not about training and testing, but what are parent contributions. We performed our analysis of the NAM datasets in two ways: (i) joint analysis of all 25 families, and (ii) independent analysis of all 25 families. The joint analysis ignores the variations among the 25 families and thus allows us to test the robustness of OSGS to familial variation. The independent analysis is limited to variations within each bi-parental cross and thus provides a good platform for demonstrating the use of OSGS.

For each analysis, we estimated correlation coefficients between the observed ($y-X\hat{b}$) and predicted trait values of $\hat{a}$, ${\hat{a}}_{1}$ and ${\hat{a}}_{2}$. $\hat{a}$ is estimated from all (A) markers, ${\hat{a}}_{1}$ is estimated from subset of markers with favorable primary (P) parent alleles, and ${\hat{a}}_{2}$ is estimated from subset of markers with favorable secondary (S) parent alleles. The relative importance of the primary and secondary parents as contributors of favorable genetic variation was quantified by the correlations between the pairs of $\hat{a}$, ${\hat{a}}_{1}$ and ${\hat{a}}_{2}$, and by the number and distribution of favorable marker effects among the two parents.

In addition, we compared the distributions of favorable primary (P) and secondary (S) marker effects. We first extracted the P and S marker effects based on the signs of rrBLUP coefficients and the favorable direction for each trait. We then converted the marker effects into absolute values and compared the two distributions using the Kolmogorov-Smirnov test as implemented in the *ks.test* function in R (R Core Team 2020). Results were shown as -log_10_(p).

To evaluate the potential of OSGS in optimizing favorable parental contributions to progeny, we compared the simulation outcomes from selection in the NAMs using OSGS and GS. To begin this simulation, we took the results from the independent analysis of the 25 NAM families. Within each family, we selected top four lines based on the estimated breeding values (EBVs) determined by OSGS and GS. In GS, the EBVs are essentially $\hat{a}$. In OSGS, we first ranked ${\hat{a}}_{1}$ and ${\hat{a}}_{2}$ such that the most favorable ${\hat{a}}_{1}$ and ${\hat{a}}_{2}$ have the highest rank value. Next, we calculated the EBVs as $rank\left({\hat{a}}_{1}\right)\cdot \omega +rank({\hat{a}}_{2})\cdot (1-\omega )$, in which ω is the selection weight for P and ranges from 0 to 1. We made all $\left(\begin{array}{c} 4\\ 2 \end{array}\right)=6$ possible crosses among these four selected lines and generated 10 double haploids (DHs) from each cross. This process was done using AlphaSim (Faux *et al.* 2016) to simulate the recombination events. We calculated the average fold change in proportions of favorable primary alleles (P) and favorable secondary alleles (S) alleles and average normalized change in EBVs before and after selection. Lastly, we compared these changes between OSGS and GS across all 25 families.

### Barley NAM population

We analyzed two polygenic traits in the HEB-25 barley NAM population: days to heading (DTH) and yield (YLD), which were respectively taken from Herzig *et al.* (2018) and Sharma *et al.* (2018). Since only raw data on DTH and YLD were provided, we calculated the least squares means of DTH and YLD for 1,420 lines based on the fixed effects of location, nitrogen treatment and year.

We also obtained the accompanying marker genotype data from Maurer *et al.* (2015), which consisted of 1,427 lines and 5,709 polymorphic markers. We removed five markers that did not map to reference genome, resulting in 5,704 markers. The markers were initially coded as 0 for homozygous elite allele, 1 for heterozygous, 2 for homozygous wild allele and 5 for non-polymorphic within family. To maintain consistency between the barley and maize NAM data, we set all the markers coded as 5 to missing and imputed these missing markers using the same method as for the maize NAM (Buckler *et al.* 2009), where any missing data were imputed as an average of two non-missing flanking markers. Markers with missing data at the start and end of each chromosome were imputed to be the same as the nearest markers. Finally, we converted the marker from 0/1/2 to -1/0/1 format.

The trait and marker data combined resulted in 1,371 lines for analysis.

### Maize NAM population

We analyzed two polygenic traits in the maize NAM population that are comparable to DTH and YLD in the barley NAM population: days to silking (DTS) and cob length (CL), which were taken from Buckler *et al.* (2009) and Brown *et al.* (2011) respectively. Similar to the barley NAM trait data, we calculated the least squares means of DTS and CL for 4,910 and 4,884 lines respectively based on the fixed effects of location, year, replication within location and block.

We also obtained the accompanying marker genotype data from McMullen *et al.* (2009) for 4,699 lines and 1,106 polymorphic markers. The marker data are fully imputed and phased, so we only converted the marker format from 0/1/2 to -1/0/1 format.

The trait and marker data combined resulted in 4,697 lines for analysis.

### Computer simulations

For comparison with the barley and maize NAMs, we simulated three bi-parental populations: (1) F_2_-derived, (2) BC_1_-derived and (3) reverse BC_1_-derived (secondary line as the recurrent parent), all of which were selfed for 4 generations prior to GS/OSGS. For each population, we simulated traits with varying ratios of favorable primary (P) and secondary (S) QTL (P:S = 50:50, 55:45, 60:40, 70:30, 80:20, 90:10) and with QTL densities of 2cM/QTL or 20cM/QTL. Within each simulation, same QTL were tested in F_2_/BC_1_/rBC_1_-derived populations.

We used rrBLUP to calculate the marker effects in the F_6_, BC_1_S_4_ and rBC_1_S_4_ generations. These were used for predicting the breeding values of each line using GS and OSGS methods. Similar weighting schemes to the NAM simulation were used here to determine the breeding values in OSGS. We crossed the top 5 lines (identified by GS/OSGS) in a half diallel and generated 20 double haploids (DHs) from each cross. Similar to the previous simulation with the NAM datasets, we compared GS and OSGS impacts on P and S proportions and EBVs over a single generation of selection. In addition, we chose the population with P:S = 60:40 and ω=0.5 and performed recurrent selection for an additional four cycles. We used the previously calculated marker effects to predict EBVs for selection purposes in all subsequent generations. Details on the selection process can be found in Figure S1. All simulations were repeated 100 times.

All simulations were performed in R 3.6.3 (R Core Team 2020), in which marker data were generated using AlphaSim v0.11.1 (Faux *et al.* 2016) and trait data were generated using custom R scripts. For all populations, we simulated diploid individuals with 10 chromosomes and 7,750 markers distributed evenly across a total genetic distance of 1,550 cM. The markers were coded as -1 for the primary parent and 1 for the secondary parent. QTL positions were randomly sampled from a uniform distribution of all markers. QTL effects for the primary and secondary parent alleles were simulated from a half-normal distribution such that the QTL marker variances are equal between primary and secondary parent alleles, and the aggregated QTL marker variance is equal to *p^-1^*, where *p* is the total number of QTL (Figure S2A). Markers selected as QTL markers were left in these analyses since their removal with such high marker density would have little effect, and our purpose is to compare the performance of OSGS and GS and not to test differences in prediction accuracy due to marker-QTL linkage. For any generation, the true breeding value of each line was calculated from its QTL marker genotypes and QTL effects, and the phenotypic trait value of each line was calculated by adding residual value drawn from a standard normal distribution with mean of 0 and variance of 1. Since we fixed the QTL marker variance and residual variance, the simulated mean trait heritabilities range from 0.40 to 0.95 depending on the proportion of favorable primary and secondary parent alleles and number of QTL markers (Figure S2B).

### Data availability

The barley NAM raw DTH, raw YLD and marker genotype datasets were downloaded from doi.org/10.5447/IPK/2017/6, doi.org/10.5447/IPK/2017/21 and Additional File 5 in doi.org/10.1186/s12864-015-1459-7 respectively. The maize NAM raw DTS, raw CL and marker genotype datasets were downloaded from the Cyverse Discovery Environment in the following folders respectively, (1) /iplant/home/shared/panzea/phenotypes/ Buckler_etal_2009_Science_flowering_time_data-090807.zip, (2) /iplant/home/shared/panzea/phenotypes/Brown_etal_2011_PLoSGenet_pheno_data-120523.zip, (3) /iplant/home/shared/panzea/genotypes/SNPs/ NAM_map_and_genos-120731.zip. All R scripts used in this manuscript can be accessed from https://github.com/cjyang-sruc/OSGS. File S1 contains all the supplemental materials. Supplemental material available at figshare: https://doi.org/10.25387/g3.12320093.

## Results

### Maize and barley NAM data analysis

OSGS is robust to the choice of GS methods as shown using three popular GS methods (rrBLUP, LASSO and BayesCπ) (Figure S3 and Table S1). There were little differences in performances across these methods, especially in $\hat{a}$ and ${\hat{a}}_{1}$. However, predictions on ${\hat{a}}_{2}$ appeared slightly more variable when LASSO is used, which is likely due to a combination of small family size and fewer favorable exotic alleles in the barley NAM. In one example, LASSO failed to identify any favorable secondary parent alleles, resulting in zero prediction from these alleles (Table S1). In some barley NAM families, the prediction accuracies of $\hat{a}$ from rrBLUP are perfect (Table S1), which suggest overfitting. However, these families also showed high accuracies with the LASSO which selects for markers by cross-validation. Nonetheless, we are not overly concerned about these perfect predictions since our interest is to show how favorable alleles can be partitioned in OSGS. Overall, since there was little difference, we focus all of our analyses on rrBLUP.

**Figure 2:**
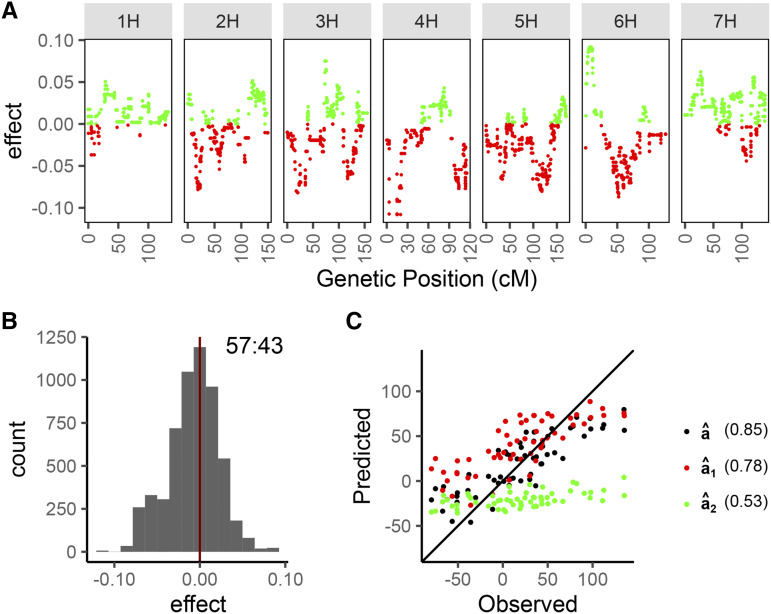
Partitioning of favorable parental alleles in OSGS. Here, we took YLD in barley NAM family 1 as an example to illustrate how the markers can be partitioned into favorable primary (P) and secondary (S) parental alleles for breeding values prediction. [A] Marker effects are plotted along the chromosomes and genetic positions, with the P alleles colored red and S colored green. [B] Distribution of marker effects shows a bias for more P (57%) than S (43%), which suggests that the recurrent parent in barley NAM has more favorable YLD allele than the donor parent 1. [C] Predicted breeding values using all markers ($\hat{a}$), P-only (${\hat{a}}_{1}$) and S-only (${\hat{a}}_{2}$) are plotted against the observed trait values, and the correlations are shown in parentheses.

Using YLD in barley NAM family 1 as an example (Figure 2), we showed the partitioning of all (A) markers into markers carrying favorable primary (P) and secondary (S) parental alleles based on their effect signs. Given our marker coding and the favorable direction of YLD, P alleles are represented by markers with negative effects and S alleles are represented by markers with positive effects. We observed an uneven distribution of P and S alleles across the genome (Figure 2A). Overall counts of P and S alleles were unequal with a slight bias toward more P alleles, as shown in Figure 2B. In our predictions using A, P or S alleles, *i.e.*, $\hat{a}$, ${\hat{a}}_{1}$ and ${\hat{a}}_{2}$, the accuracies decreased in the order of A, P and S (Figure 2C). Since P and S alleles are subsets of A, the prediction accuracies from either P or S can never exceed A’s. Prediction accuracies for all families and traits can be found in Table S1.

**Figure 3:**
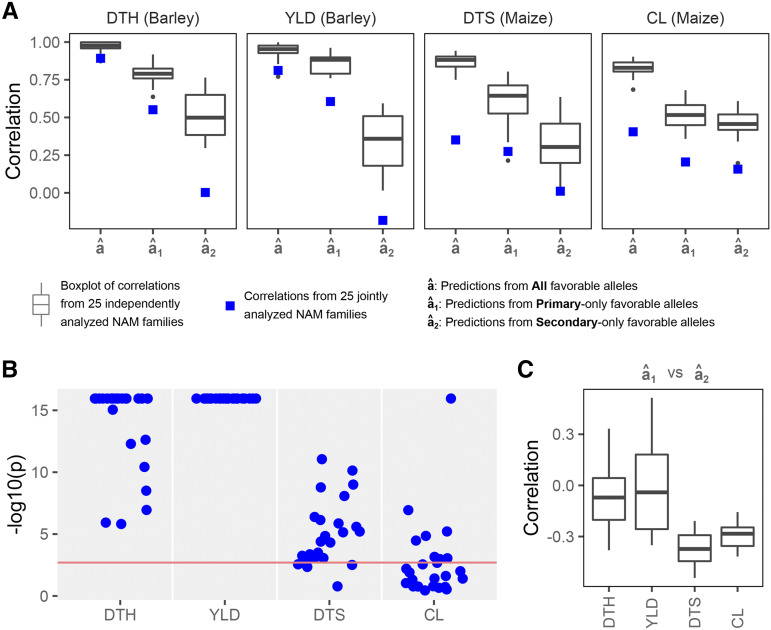
Prediction accuracies and marker effect distributions across all 25 NAM families. [A] Prediction accuracies of $\hat{a}$, ${\hat{a}}_{1}$ and ${\hat{a}}_{2}$ are shown as the correlations between the predicted and observed trait values from the joint and independent analyses of 25 NAM families. [B] P and S distributions estimated from the independent analyses were tested using Kolmogorov-Smirnov test and the results are shown as -log_10_(p). The Bonferroni adjusted threshold of *P* = 0.05/25 is shown as a red horizontal line. [C] Correlations between ${\hat{a}}_{1}$ and ${\hat{a}}_{2}$ represent the potential constraints when selecting for both P and S markers in OSGS, although the lack of strong negative correlations suggests these are small.

Between our joint and independent analyses of 25 NAM families, we found higher accuracies, but varying in degrees, in the independent analysis over joint analysis across all predictions (Figure 3A). In barley NAM, the discrepancies between the joint and independent analyses are less pronounced in $\hat{a}$ and ${\hat{a}}_{1}$ than ${\hat{a}}_{2}$. In maize NAM, the discrepancies are relatively similar across $\hat{a}$, ${\hat{a}}_{1}$ and ${\hat{a}}_{2}$. This observation is likely explained by how the NAMs were generated as the barley NAMs are BC_1_-derived and the maize NAMs are F_2_-derived. On average, the recurrent (common) NAM parent contributes approximately three-quarter in the BC_1_ genome but only half in the F_2_ genome. Unlike the recurrent parent, the donor parents are distinct and thus likely possess allelic variations, as shown in previous GWAS analyses (Buckler *et al.* 2009; Brown *et al.* 2011; Herzig *et al.* 2018; Sharma *et al.* 2018). Therefore, higher proportion of recurrent parent results in better predictions in the joint analysis.

The order of $\hat{a}$, ${\hat{a}}_{1}$ and ${\hat{a}}_{2}$ accuracies remained similar in all analyses, although the accuracy gaps among $\hat{a}$, ${\hat{a}}_{1}$ and ${\hat{a}}_{2}$ differed when compared across traits (Figure 3A). Accuracy gaps between $\hat{a}$ and ${\hat{a}}_{1}$ are smallest in YLD than the others, while accuracy gaps between ${\hat{a}}_{1}$ and ${\hat{a}}_{2}$ are largest in YLD, intermediate in DTH and DTS, and smallest in CL. This observation can be partly attributed to the NAM population types as previously suggested, however, a more important factor is likely the difference in distributions of P and S alleles across traits (Figure 3B). YLD showed the strongest difference between P and S distributions, followed by DTH, DTS and CL. Therefore, the greater the imbalance between P and S distributions, the smaller the gap between $\hat{a}$ and ${\hat{a}}_{1}$ as $\hat{a}$ is largely predicted by P.

Distribution of marker effect estimates can inform about the proportion of favorable alleles contributed by each parent (Figure 2B, Figure S4-7). Late flowering in temperate environment (northern Europe) and high yield are favored in spring barley, while early flowering and large ear size are favored in maize, thus favorable DTH, YLD and CL are represented by positive marker effects and favorable DTS is represented by negative marker effects. Across all traits, we found variable proportions of favorable alleles (Table S1). The means and ranges of P proportions across all 25 families estimated from rrBLUP were 0.52 and 0.43 – 0.62 for barley DTH, 0.63 and 0.48 – 0.78 for barley YLD, 0.56 and 0.43 – 0.65 for maize DTS and 0.51 and 0.43 – 0.59 for maize CL. In barley, we found that the primary (elite) parent had slightly more favorable DTH alleles but many more favorable YLD alleles than the secondary (exotic) parents. In maize, we found that the primary parent had more favorable DTS alleles but about equal favorable CL alleles compared to the secondary parents. Provided that a trait is polygenic, results here suggested that the distribution of marker effects can be used as a reasonable approximation to the true proportions of favorable QTL.

In addition, most of the P and S distributions were significantly different, especially in the barley NAM population (Figure 3B). By comparing the P and S distributions for each trait and family using a Kolmogorov-Smirnov test, we found that all 25 barley NAM families but only about half of the maize NAM families had significant differences. The strongest difference in the distributions was observed in barley YLD, followed by barley DTH, maize DTS and maize CL. The distributions of P and S are more likely to be different in elite-exotic crosses (barley NAM) than elite-elite crosses (maize NAM). While rrBLUP assumes a single normal distribution of marker effects, the model is robust to the violation of the assumption given the good prediction accuracies from P and S.

There were weak negative correlations between ${\hat{a}}_{1}$ and ${\hat{a}}_{2}$ across all four traits (Figure 3C). While a strong positive correlation between the two would be ideal for selection, the lack of strong negative correlations implies that we can still select for both P and S without any severe constraints. To do so, we can apply index selection based on the ranks of ${\hat{a}}_{1}$ and ${\hat{a}}_{2}$ by treating the two predictions as two separate traits.

**Figure 4:**
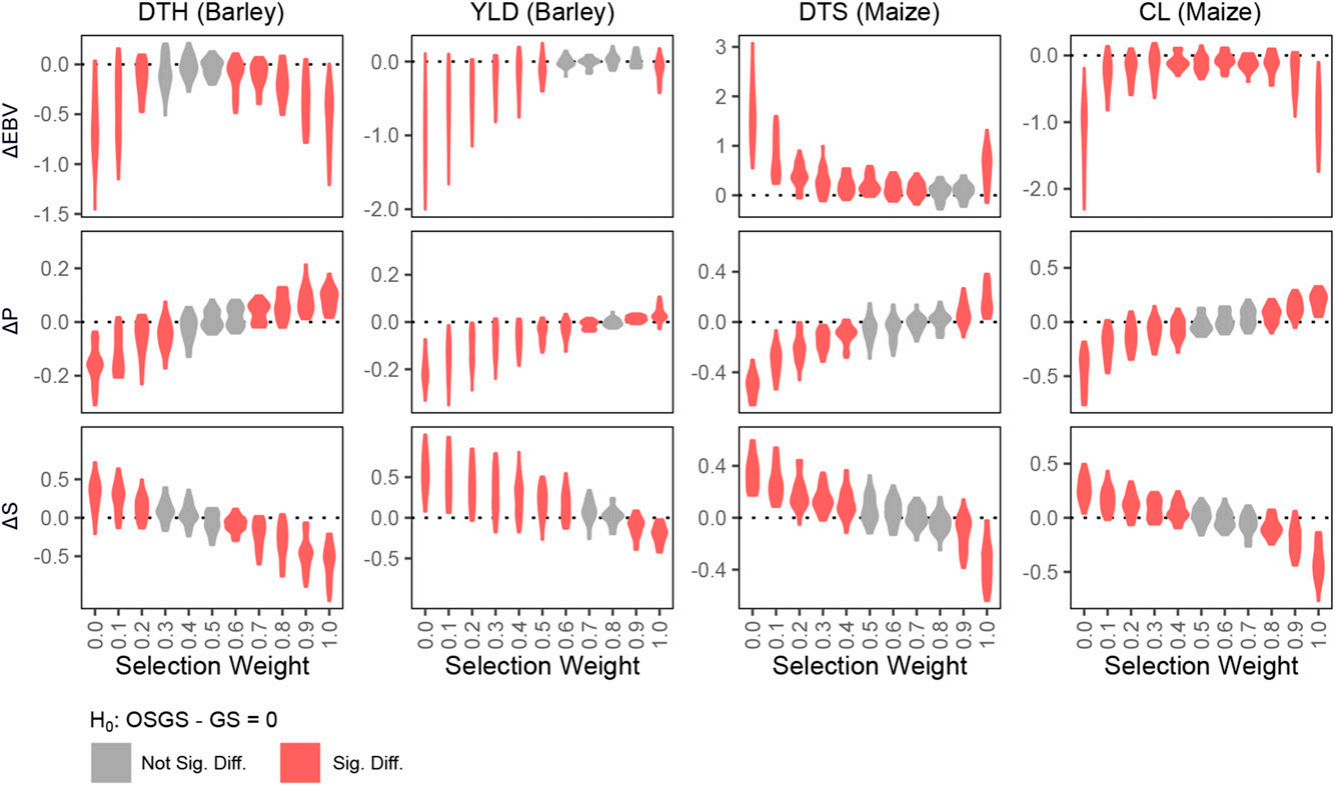
Performance comparison of OSGS under different ω against GS in NAMs. We simulated a single generation of selection under OSGS/GS and evaluated the change in estimated breeding value (ΔEBV), P proportion (ΔP) and S proportion (ΔS). ΔEBV is calculated as $\left(\mu_{EBV,OSGS}-\mu_{EBV,GS}\right)/\mu_{EBV,0}$, where $\mu_{EBV,OSGS}$ is the mean EBV of a family selected under OSGS, $\mu_{EBV,GS}$ is the mean EBV of a family selected under GS, and $\mu_{EBV,0}$ is the mean EBV of the initial (pre-selection) NAM family. Similarly, ΔP is calculated as $\left(\mu_{P,OSGS}-\mu_{P,GS}\right)/\mu_{P,0}$ and ΔS is calculated as $\left(\mu_{S,OSGS}-\mu_{S,GS}\right)/\mu_{S,0}$. Significance is determined by *t*-test with Bonferroni correction (*P* = 0.05/25).

To evaluate the usefulness of OSGS in introgressing exotic alleles in a pre-breeding context, we simulated a single generation of selection on all four traits (Figure 4). In terms of estimated breeding values (EBVs), OSGS did not outperform GS in any of the tested selection weights (ω). However, OSGS can increase or decrease P and S in comparison to GS. As ω decreased, P decreased and S increased, and vice versa. Based on these results, the ideal selection weights would be those that maximize the increase in S and minimize the EBV gap. Across all four traits, ω of 0.4 to 0.6 appeared reasonable for efficient introgression of exotic alleles in pre-breeding programs.

### Simulated data analysis

**Figure 5:**
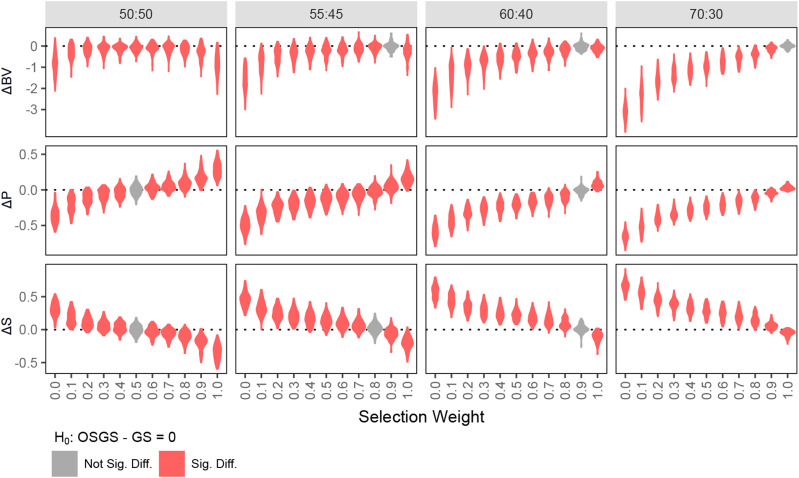
Performance comparison of OSGS under different ω against GS in simulated F_2_-derived populations. We applied a single generation of selection under OSGS/GS on 100 simulated F_2_-derived populations (2cM/QTL) and evaluated the change in true breeding value (ΔBV), P proportion (ΔP) and S proportion (ΔS). Significance is determined by *t*-test with Bonferroni correction (*P* = 0.05/25).

First, we evaluated the performance of OSGS under different proportion of P and S QTL and ω (Figure 5, S8 and S9), and found that it can be optimized based on the proportion of P and S. In the case of P:S = 50:50, OSGS with ω of 0.5 resulted in similar true breeding values (BV) and P:S proportions to GS. As the proportion of P:S increases, a slight increase in ω can minimize the BV gap between OSGS and GS, and still maintain a higher S proportion in OSGS than GS. Given that the proportions of estimated P and S marker effects reasonably approximated the true proportion of P and S QTL (Figure S10), we can adjust ω in OSGS according to the estimated P and S proportions.

**Figure 6:**
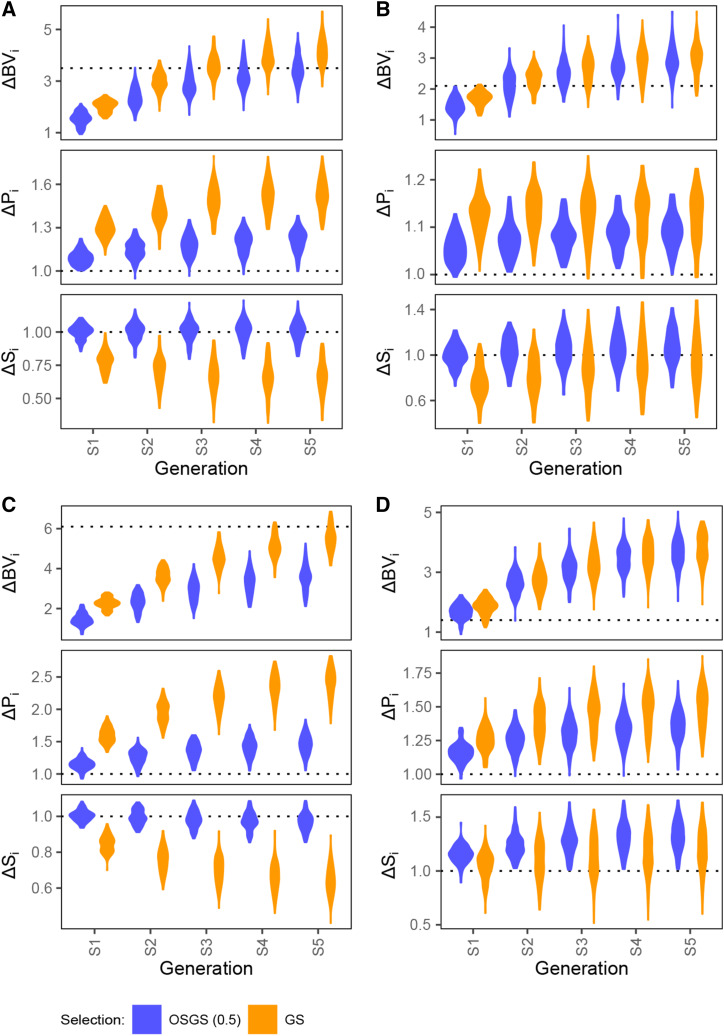
Performance comparison of OSGS against GS under recurrent selection. We applied five generations of recurrent selection under OSGS/GS on 100 simulated F_2_, BC_1_ and rBC_1_-derived populations with P:S of 60:40. We showed the change in true breeding value ($\Delta BV_{i}$), P proportion ($\Delta P_{i}$) and S proportion ($\Delta S_{i}$), where i is either OSGS with ω=0.5 or GS. $\Delta BV_{i}$ is calculated as $\left(\mu_{BV,i}-\sigma_{BV,0}\right)/\mu_{BV,0}$, $\Delta P_{i}$ as $\mu_{P,i}/\mu_{P,0}$ and $\Delta S_{i}$ as $\mu_{S,i}/\mu_{S,0}$. [A] F_2_ with 2cM/QTL. [B] BC_1_ with 2cM/QTL. [C] rBC_1_ with 2cM/QTL. [D] F_2_ with 20cM/QTL.

Comparing across F_2_, BC_1_ and rBC_1_-derived populations, OSGS is best performed in an F_2_ population as it begins with an equal proportion of primary and secondary parent alleles (Figure 6 and S11, Table 2). F_2_ population provides a good starting ground for OSGS to elevate S proportion while keeping the BV gap with GS low. In a BC_1_ population, there is already a bias in the population toward primary parent alleles as the population has 75% primary parent alleles and 25% secondary parent alleles on average. While it is possible to minimize BV gap between OSGS and GS, there is little gain in S over multiple generations of recurrent selection. On the other hand, in a rBC_1_ population, the BV gap is too large to compensate for the gain in S. From a different perspective in the absence of OSGS, one is better off applying GS in a BC_1_ over an F_2_ population as it achieves higher breeding values faster (Figure 6 and S11, Table 2) without losing much S in the process.

**Table 2:** Consequences of OSGS/GS on the proportions of favorable alleles and breeding values. Using P:S ratio of 60:40 as an example, we compared the mean proportions of favorable primary (P) and favorable secondary (S), and the true BV after five generations of recurrent selection. First two rows are the parents, third row is the pre-selected population, and the remaining are selected populations. P and S in generation 0 are essentially weighted means of P and S in the parents where the weights are the mean proportion of parental markers. For example, in BC_1_, *P* = 0.75 × 0.60 = 0.45 and S = 0.25 × 0.40 = 0.10

Gen	ω	F_2_			BC_1_			rBC_1_		
		P	S	BV	P	S	BV	P	S	BV
P	—	0.60	0.00	4.40	0.60	0.00	4.40	0.60	0.00	4.40
S	—	0.00	0.40	−4.40	0.00	0.40	−4.40	0.00	0.40	−4.40
0	—	0.30	0.20	0.00	0.45	0.10	2.19	0.15	0.30	−2.18
5	0.0	0.11	0.35	−1.41	0.25	0.26	1.42	0.03	0.39	−3.48
5	0.1	0.21	0.30	1.27	0.36	0.20	3.82	0.09	0.36	−1.76
5	0.2	0.28	0.26	3.02	0.42	0.16	4.85	0.14	0.34	−0.18
5	0.3	0.31	0.24	3.57	0.45	0.14	5.22	0.17	0.32	0.63
5	0.4	0.34	0.21	4.19	0.47	0.12	5.30	0.20	0.30	1.22
5	0.5	0.36	0.20	4.40	0.49	0.11	5.43	0.22	0.29	1.70
5	0.6	0.39	0.18	4.74	0.51	0.09	5.39	0.24	0.28	2.17
5	0.7	0.41	0.17	5.01	0.52	0.08	5.50	0.27	0.26	2.55
5	0.8	0.44	0.14	5.09	0.54	0.06	5.36	0.30	0.24	3.09
5	0.9	0.49	0.10	5.14	0.57	0.03	5.00	0.36	0.20	3.63
5	1.0	0.54	0.05	4.66	0.59	0.01	4.55	0.43	0.14	3.60
5	GS	0.46	0.13	5.29	0.50	0.10	5.48	0.36	0.19	3.83

Lastly, comparing between QTL density of 2cM/QTL and 20cM/QTL, there is more merit to using OSGS when the number of QTL are large (Figure 6A, 6D and S11). In the case where the QTL density is low (20cM/QTL), there is little difference between GS and OSGS (Figure 6D and S11) aside from OSGS is slightly better in increasing the S proportion. However, as we increased the QTL density to 2cM/QTL, we found that OSGS was able to keep the balance between favorable primary and secondary parent alleles throughout selection, while GS resulted in a larger discrepancy (Figure 6A and S11). This highlights the issue with GS in an elite-exotic population as few exotic alleles manage to enter the final breeding population. OSGS can be used to address this issue.

## Discussion

In the recent years, there has been a growing interest in exploring ways for efficient introduction of novel genetic variation from exotic germplasm like landraces and wild relatives into modern breeding populations (Mascher *et al.* 2019). Even in elite crosses, current selection practices can be strongly biased in favor of one parent (Fradgley *et al.* 2019), and linkage drag may limit the potential for favorable alleles to be selected from the phenotypically weaker of the two genomes. To circumvent this problem, Gorjanc *et al.* (2016) suggested an approach to create improved lines from purely exotic materials prior to crossing with the elite materials. Samayoa *et al.* (2018) suggested a slightly different approach where the exotic improvement is only performed on adaptation-related traits. Alternatively, Han *et al.* (2017) formulated a method to identify candidate exotic lines for introgressing small numbers of favorable exotic alleles into elite populations. Allier *et al.* (2020) further extended this approach for introgressing a larger number of favorable exotic alleles by identifying exotic candidates with higher ratios of favorable over unfavorable alleles. In a slightly different approach, Allier *et al.* (2019) proposed the usefulness criterion parental contribution (UCPC) as a metric that combines both the usefulness criterion (Schnell and Utz 1975) and parental genomic contributions in identifying exotic materials for crossing with elite populations. Ru and Bernardo (2019 and 2020) proposed introgressing linkage groups over QTL via targeted recombination.

While these approaches seem promising, none of them directly addresses the issues of genomic selection in elite-exotic populations. These approaches focus on identifying the best possible exotic line for crossing, and none attempts to improve the exotic introgression potential after crossing exotic and elite lines. Improvement on solely exotic lines (Gorjanc *et al.* 2016) may risk selecting for favorable alleles that are already present in elite populations. Selecting for exotic lines with the best combination to the target elite lines (Han *et al.* 2017; Allier *et al.* 2019; Allier *et al.* 2020) likely requires accurate predictions on the crosses performances, which calls for large training population and/or close relationships among the selected lines that may not be available.

Here, we propose using OSGS as a generalized framework for partitioning favorable trait contributions among parents. When applied on a single elite-exotic cross population, high prediction accuracies will be possible without requiring a large sized population for phenotyping (Brandariz and Bernardo 2019). This subsequently allows us to partition these predictions into favorable primary and secondary parental contributions with high confidence. OSGS is flexible with respect to the choice of the exotic genome and is complementary to any of the previously described approaches to accommodate those selected exotic lines. In addition, we have demonstrated that OSGS works using the barley and maize NAMs, furthering the potential of community-generated genetic resources as potent breeding tools. Moreover, Bernardo (2009) and our results suggest that it is likely better to use F_2_-derived NAMs to backcross-derived NAMs for this purpose.

In general, OSGS is robust to the choice of a statistical method and should work for other untested methods provided marker effects can be estimated and partitioned into two or more classes. However, one might consider models that are better suited for the presumed trait genetic architectures. For example, LASSO might be a better option for traits regulated by few QTL since LASSO reduces the effects of most markers to zero.

In this paper we have shown that OSGS can maintain the balance between favorable primary and secondary parent allele proportions over several generations of selection. Hence, OSGS may also play a similar role in optimal contribution selection initially suggested by Meuwissen (1997). Optimal contribution selection aims to maintain genetic diversity in a population under selection by penalizing the estimated breeding values with relationships among selected individuals (Woolliams *et al.* 2015). In genomic setting this penalty is based on genomic relationships identified from all markers, which does not distinguish between favorable primary and secondary parent alleles. Therefore, OSGS can be complementary to optimal contribution selection as we could partition the kinship matrix into two matrices based on markers carrying favorable primary or secondary parent allele effects. Similar approach has been advocated for optimal contribution selection in rare breeds of livestock in the presence of introgression from cosmopolitan breeds (Wang *et al.* 2017a, 2017b and 2019).

There are several applications of OSGS remaining to be explored. We found that the distributions of favorable primary and secondary parent effects are different, especially in elite-exotic crosses. This is expected because of the joint action of selection and drift during and after species domestication. OSGS may provide an approach to studying this effect by comparing distributions across populations and species. The application of OSGS could be extended to multi-parental crosses using predictions based on identity-by-descent relationships due to originating parents. Multi-parent populations based on two or more elite lines and a single exotic parent are already in use in pre-breeding (Hao *et al.* 2019; Singh *et al.* 2018). There is a strong risk that phenotypic or genomic selection in these populations will discriminate against favorable alleles carried by the exotic parent to an even greater extent than we have shown in bi-parental populations (see also simulations by Gorjanc *et al.* 2016).

There might also be merits in combining OSGS with other approaches. For example, we can combine the parent selection approaches of Allier *et al.* (2020) with OSGS. This may be particularly useful for breeding programs that attempt to use elite and exotic lines with high performance gaps in the traits of interest. In addition, OSGS can be extended to work with gametic variance-based selection (Bijma *et al.* 2020) by maintaining a balance in the parental contributions on gametic variance.

Lastly, the most promising application of OSGS may be its extension to multi-trait selection. This could be especially useful in elite-exotic crosses where the traits are not unanimously favorable in the elite lines. For example, the exotic parent may carry most favorable alleles for abiotic or biotic stress resistance, but the elite parent mostly for productivity traits.
